# Demonstrating synchronization of isorhythmic dissociation with stress testing

**DOI:** 10.1002/ccr3.2591

**Published:** 2019-12-05

**Authors:** John‐Ross David Clarke, Faheem Ul Haq, Saurabh Ranjan, Stuart Zarich

**Affiliations:** ^1^ Department of Internal Medicine Yale‐New Haven Health/Bridgeport Hospital Bridgeport CT USA; ^2^ Department of Cardiology Yale‐New Haven Health/Bridgeport Hospital Bridgeport CT USA

**Keywords:** AV dissociation, escape rhythm, Isorhythmic dissociation

## Abstract

There are a variety of causes of atrial and ventricular dyssynchrony. The mechanism underlying the arrhythmia is usually a guide to further management. This case highlights the key distinguishing features of more benign etiologies.

## Graphical abstract

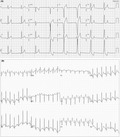

## 1 INTRODUCTION

A 26‐year‐old gentleman presented with intermittent, nonradiating, left‐sided chest discomfort of four days’ duration—without exertional or positional variation. His symptoms had resolved prior to presentation. He had a history of adolescent‐onset hypertension and used marijuana recreationally.

**Figure 1:**
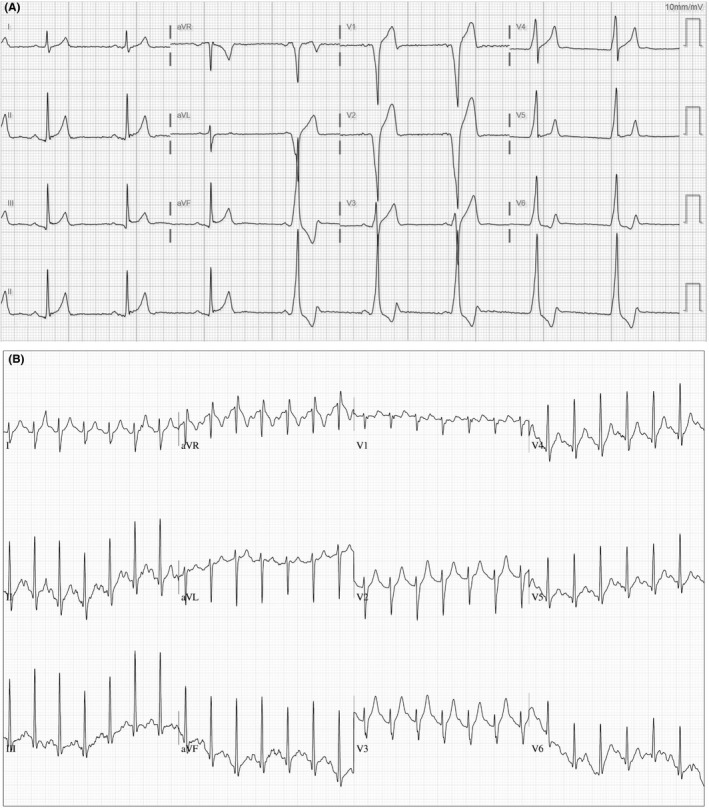
(Panel A) Resting electrocardiogram demonstrating normal AV conduction from sinus impulses for the first three beats. Isorhythmic dissociation occurs thereafter, with P waves ‘wandering’ before, within or after the QRS complexes at about the same rate. (Panel B) Synchronization of AV conduction without arrhythmia or ischemia at peak exercise

Physical examination was noncontributory. ECG is shown in Figure 1 (panel A).

What is the diagnosis?

- Slow ventricular tachycardia
- AV reentrant tachycardia
- Isorhythmic AV dissociation
- Complete AV block.

## 2 DISCUSSION AND OUTCOMES

Our patient's resting ECG (Figure 1 panel A) initially demonstrates sinus bradycardia with gradual slowing. After three beats, an automatic focus from the right bundle or RV myocardium escapes. Transthoracic echocardiogram showed normal ejection fraction and mild concentric hypertrophy without regional wall motion abnormalities. Serum laboratory testing was normal. The decision was made to proceed with exercise‐ECG testing to evaluate for chronotropic competence and whether high‐degree AV conduction delays occurred with stress. Stress testing demonstrated AV synchronization without symptoms or ischemic changes (Figure 1 panel B). He has not had recurrence of symptoms nor required further evaluation.

Isorhythmic AV dissociation is uncommon in the general population but is often an incidental finding in young individuals and up to one‐fifth of endurance athletes—felt to be related to excessive vagal tone.^1^ It is characterized by occasional atrioventricular conduction—distinguishing it from complete AV block.^2^ Isorhythmic dissociation usually carries a good prognosis. It seldom requires further investigation unless associated with symptoms of myocardial ischemia, functional limitation, or syncope.

## CONFLICT OF INTEREST

None declared.

## AUTHOR CONTRIBUTION

JC, FU, and SR: wrote the initial draft. SZ: provided expertise in image interpretation. All authors: participated in collecting patient data (images and clinical history), reviewing the literature, interpretation of clinical findings, critical revision of the manuscript for important intellectual content, and approval of the final version.
